# Potential predictors of immunotherapy in small cell lung cancer

**DOI:** 10.3389/pore.2023.1611086

**Published:** 2023-05-03

**Authors:** Valeria Skopelidou, Jan Strakoš, Jozef Škarda, Milan Raška, Leona Kafková-Rašková

**Affiliations:** ^1^ Institute of Molecular and Clinical Pathology and Medical Genetics, University Hospital Ostrava, Ostrava, Czechia; ^2^ Institute of Molecular and Clinical Pathology and Medical Genetics, Faculty of Medicine, University of Ostrava, Ostrava, Czechia; ^3^ Department of Clinical and Molecular Pathology, Faculty of Medicine and Dentistry, Palacký University Olomouc, Olomouc, Czechia; ^4^ Department of Immunology, Faculty of Medicine and Dentistry, Palacký University Olomouc, Olomouc, Czechia; ^5^ Department of Immunology, University Hospital Olomouc, Olomouc, Czechia

**Keywords:** tumor microenvironment, PD-L1 expression, small cell lung cancer, mutational burden, molecular subtyping

## Abstract

Lung cancer is one of the leading causes of cancer-related deaths worldwide, with small cell lung cancer (SCLC) having the worst prognosis. SCLC is diagnosed late in the disease’s progression, limiting treatment options. The most common treatment for SCLC is chemotherapy. As the disease progresses, immunotherapy, most commonly checkpoint inhibitor medication, becomes more important. Efforts should be made in the development of immunotherapy to map specific biomarkers, which play a role in properly assigning a type of immunotherapy to the right cohort of patients, where the benefits outweigh any risks or adverse effects. The objective of this review was to provide a thorough assessment of current knowledge about the nature of the tumor process and treatment options for small cell lung cancer, with a focus on predictive biomarkers. According to the information obtained, the greatest potential, which has already been directly demonstrated in some studies, has characteristics such as tumor microenvironment composition, tumor mutation burden, and molecular subtyping of SCLC. Several other aspects appear to be promising, but more research, particularly prospective studies on a larger number of probands, is required. However, it is clear that this field of study will continue to expand, as developing a reliable method to predict immunotherapy response is a very appealing goal of current medicine and research in the field of targeted cancer therapy.

## Introduction

Lung cancer is one of the most common causes of cancer-related deaths not only in the Czech Republic, but also worldwide. With more than half of patients having an advanced stage of cancer at the time of diagnosis, where aggressive surgical treatment is no longer an option, there are over 2 million new cases, and 1.76 million fatalities reported each year. Among the significant etiological factors are active smoking, passive smoking, inhalation of radon or asbestos and exposure to ionizing radiation (1–5).

**FIGURE 1:**
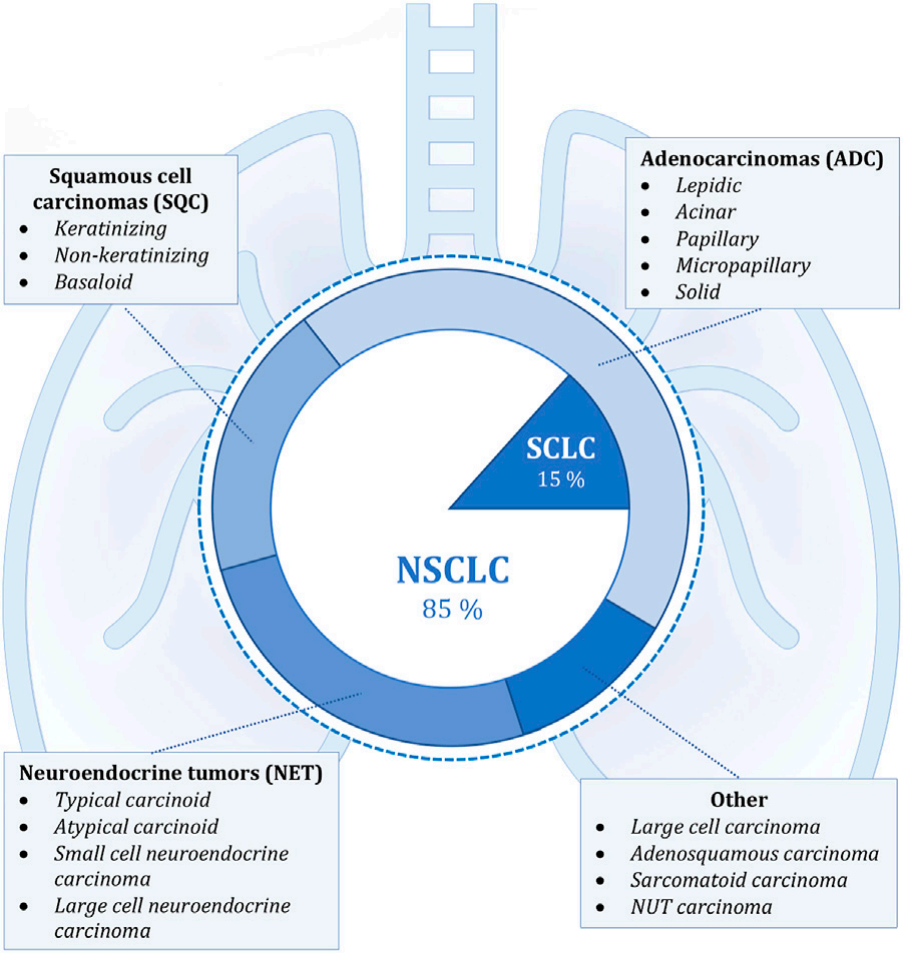
Shows the classification of lung cancer according to histopathological morphology. The internal simpler division corresponds to the basic categorization into non-small cell (NSCLC) and small cell lung cancer (SCLC). The external, more detailed classification into individual groups corresponds to the new classification according to the WHO, which was implemented in 2021. The most frequently occurring malignant lesions are listed in the individual categories (4, 6–8).

Lung carcinomas represent a heterogeneous group of malignant epithelial tumors with a wide spectrum of clinicopathological features (Figure 1). These cancerous tumors can develop from the parenchyma of the lung or the bronchial tissue. It is not always feasible to accurately identify the origin of tumor cells, and it is also not necessary for future diagnosis or treatment. The key role in the diagnostic process is played by the identification of the histological type of tumor cells, which is done through the evaluation of cytological (including cytoblocks) or histological samples. Histopathological evaluation to determine the exact diagnosis can be based on several types of samples—the most common is bronchoscopic, needle (namely CT-guided transthoracic or transbronchial needle aspiration—depending on the location of the lesion) or surgical biopsy (surgical resection specimens), which also includes thoracoscopy, excisional wedge biopsy, lobectomy, or pneumonectomy. The simpler classification involves dividing lung tumors into two subgroups, namely non-small cell (NSCLC; approximately 85% of all tumors) and small cell (SCLC; the remaining 15%) lung cancers. This basic division roughly expresses the biological nature of the cancer tissue (higher growth rate and early metastasis in SCLC) and was previously sufficient for therapeutic purposes, since the same treatment procedures were used in clinical practice for all non-small cell carcinomas regardless of further histopathological subtyping. However, it’s diametrically opposed from the therapeutic strategy for small cell carcinomas (these are mainly non-surgical procedures). More emphasis is currently being placed on improving morphological classification as new and more specialized anti-tumor therapies, particularly immunotherapy or biological treatment, develop. Molecular genetic subtyping methods are also becoming more significant (2, 3, 6, 9–13).

**FIGURE 2:**
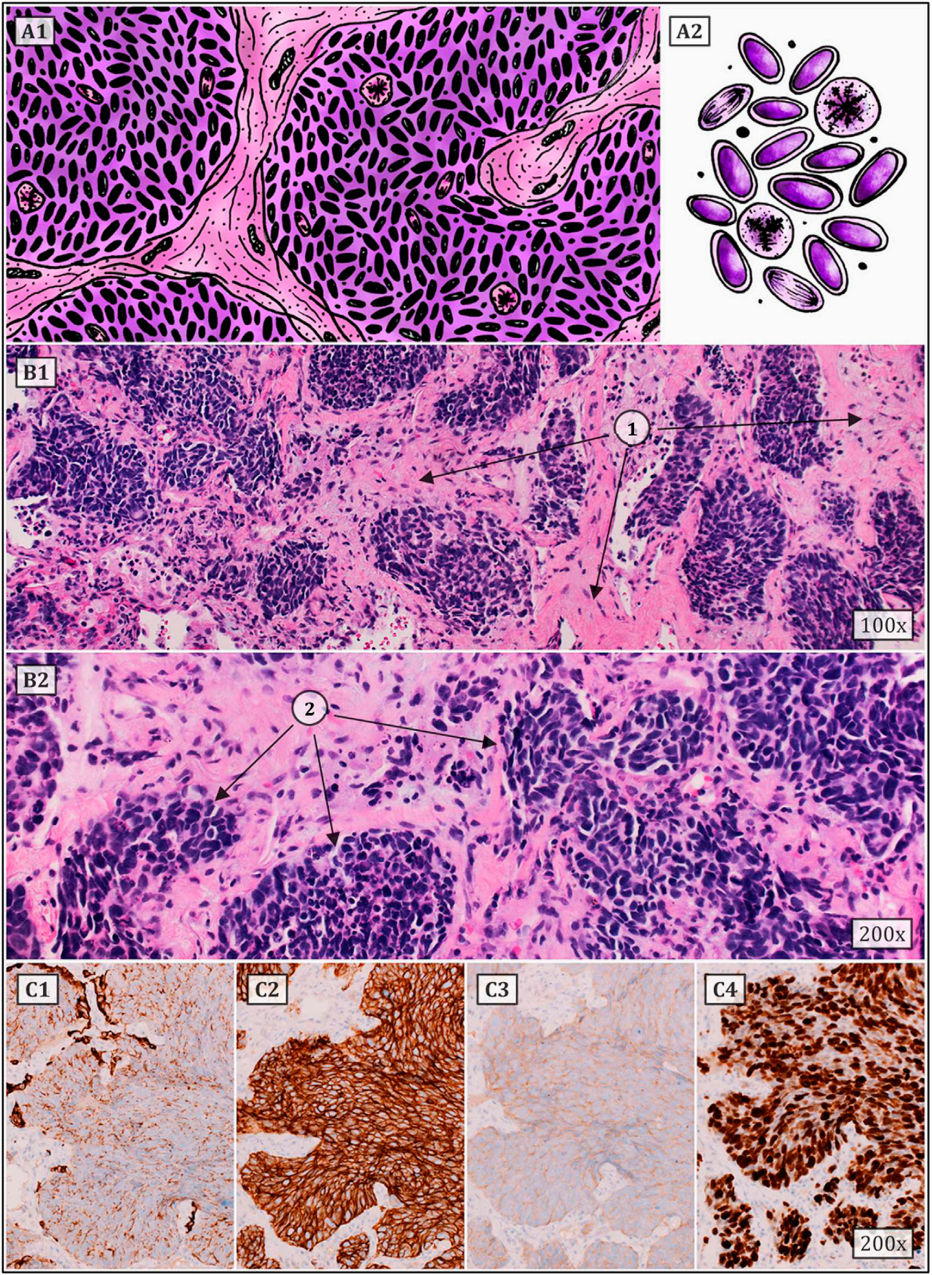
Typical SCLC consists of small cells (they can be round, oval or spindle-shaped) with a faint rim of cytoplasm, weakly defined cell border, finely granular nuclear chromatin, and indistinct or absent nucleoli. Numerous mitoses (also atypical) are common. Architectural patterns include nesting, trabeculae, peripheral palisading, and rosette formation. **(A1,2)** show a schematic drawing of SCLC. **(B1,2)** represent classic HE staining (1 – tumor stroma, 2 – tumor cell population). **(C1–4)** correspond to immunohistochemistry staining, i.e., **(C1)** – cytokeratin 7, **(C2)** – CD56, **(C3)** – synaptophysin and **(C4)** – proliferative Ki67 index (approx. 80%) (6, 12, 14–17).

Small cell lung carcinoma (SCLC) is defined as a malignant epithelial tumor consisting of small round, oval, or spindle-shaped cells with a faint rim of cytoplasm, weakly defined cell border, finely granular nuclear chromatin, and indistinct or absent nucleoli (Figure 2). Typical SCLC involves only small cells (90% of cases), other types are classified as combined disease where the tumor also contains large cell components. It is considered to be one of the most aggressive malignant tumors ever. This form of lung cancer, which mainly affects heavy smokers, is frequently discovered in its advanced stages, which is linked to a greater early mortality rate than in NSCLC. The five-year survival rate for patients diagnosed with SCLC ranges between 2.2% and 3.7%, which is significantly lower than the 10.8%–14% range for non-small cell carcinoma. Although the suspicion of small cell carcinoma already arises from the results of imaging methods or the patient’s symptoms, a pathological or cytological examination is required to confirm the diagnosis. Samples from the primary tumor, lymph nodes or metastatic locations should be taken by bronchoscopic biopsy or fine needle aspiration. Immunohistochemical methods must be used to confirm equivocal and complicated cases, with testing of neuroendocrine markers such as chromogranin, synaptophysin, and CD56 being among the most useful. Less than 10% of all small cell carcinomas are negative for all of the aforementioned markers. SCLCs are also positive for TTF-1 in up to 90% of cases. Testing the expression of epithelial markers such as cytokeratins is also appropriate, as they are observed in many small cell carcinomas and help distinguish them from lymphomas or other small round cell tumors. The proliferative Ki-67 index ranges between 80 and 100 percent, which also serves to differentiate SCLC from carcinoids. Approximately 90% of all small cell carcinomas are located centrally. With the gradual expansion of the tumor, the surrounding bronchi are frequently compressed. Two additional pathways for tumour spread are the lymphangitic network in the lungs and regional lymph nodes (14–17).

**FIGURE 3:**
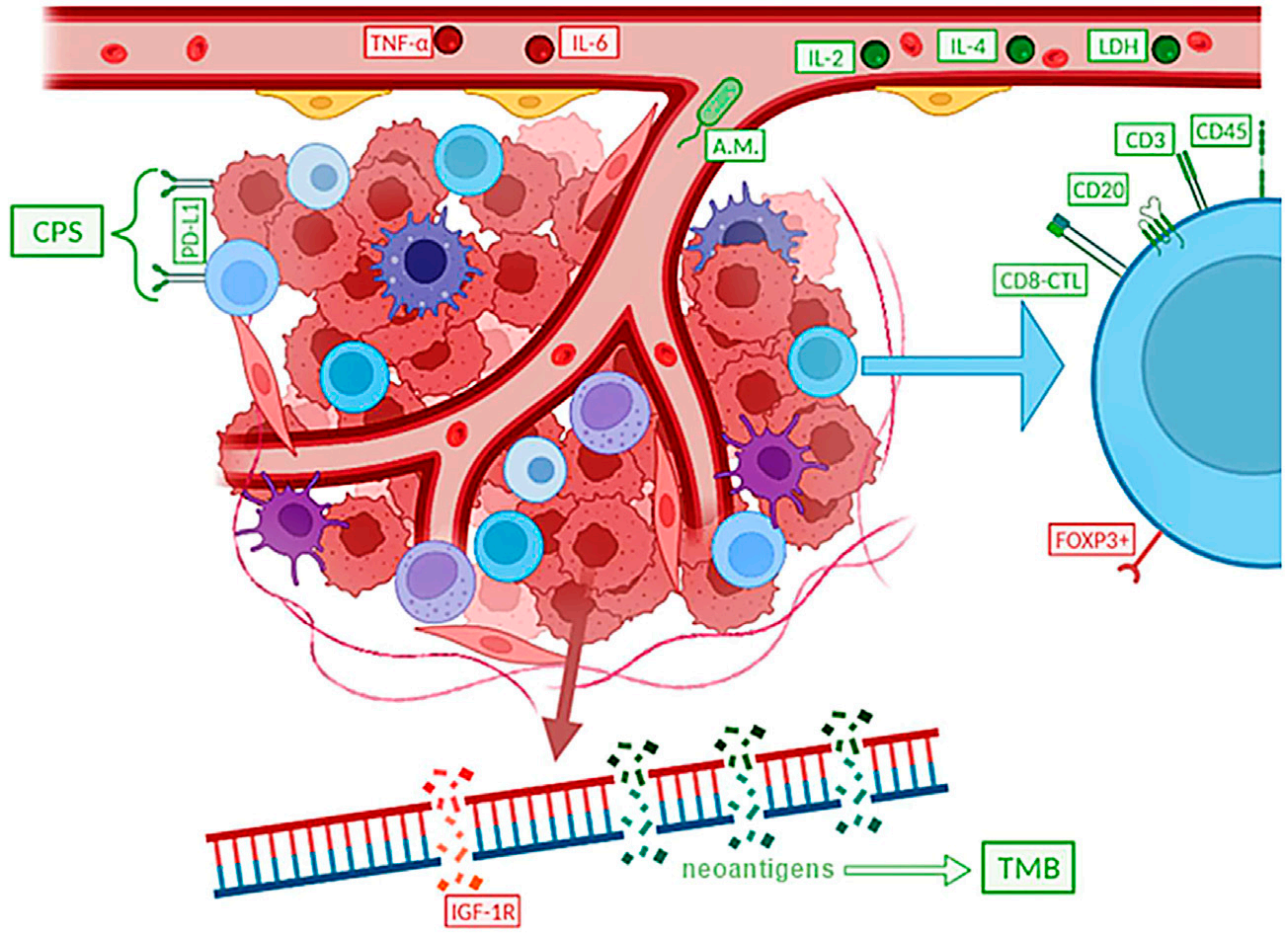
Provides a flowchart that was approved by the FDA in 2021. Figure depicts the only approved indication for immunotherapy so far, in the treatment of advanced stage SCLC. An important differentiating factor is PS, the so-called performance status, which corresponds to the patient’s overall condition (according to WHO: 1 – full health, 5 – dead) (18–23).

Several treatment options are currently available for small cell lung cancer. While it is critical to act in the patient’s best interest, it is also necessary to carefully weigh the risks and benefits of any therapy in order to obtain an effective therapeutic effect. Chemotherapy is the most common treatment for SCLC, at least in the present. This is regarded as basic therapy by the American Cancer Society (ACS) recommendations as of 2021. However, the administration of chemotherapeutic drugs is associated with several side effects that can significantly limit the patient’s recovery, with the most common being hair loss, oral ulcers, weight changes, nausea or vomiting, and constipation or diarrhoea dominating gastrointestinal problems. The negative effect is also observed in the bone marrow, due to which symptoms of fatigue (anaemia), bleeding (thrombocytopenia) and infection (leukopenia) appear, all of which often improve once the treatment is stopped. SCLC is a type of tumor that frequently has numerous metastatic foci at the time of its diagnosis, and thus curative surgery is considered in less than 1 in 20 patients in whom the oncological disease was detected at the stage of a solitary operable centre without metastases. The indication of appropriate treatment is also influenced by the cancer’s overall stage. If the SCLC is limited, then concurrent chemoradiation is considered. However, if the stage of the tumor is advanced, radiation therapy is abandoned, and this is where, in addition to chemotherapy, immunotherapy also comes into play (Figure 3). Small cell lung carcinoma’s treatment choices are rather restricted, as can be observed, thus efforts to enhance and streamline therapeutic approaches are necessary. The focus should be on immunotherapy techniques, which are now only used for advanced stages of the illness (18, 24–26).

## Potential predictors of immunotherapy in small cell lung cancer

**FIGURE 4:**
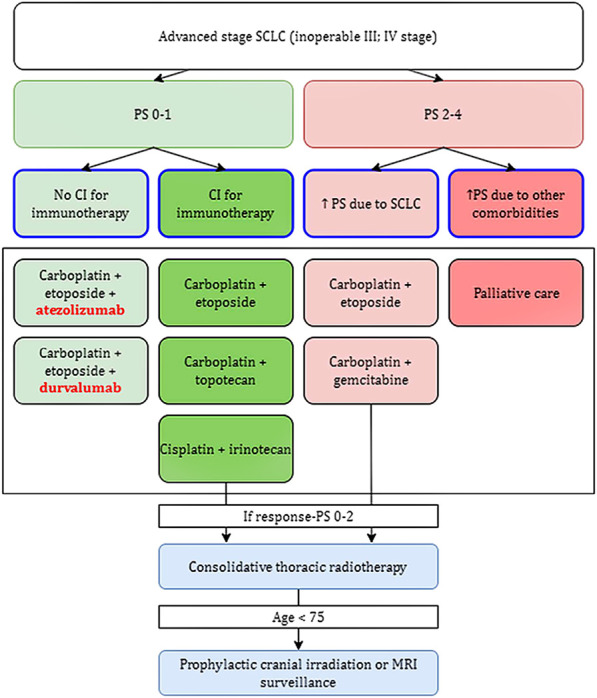
Simply summarizes potential predictive biomarkers of immunotherapy in SCLC, with negative predictive factors marked in red and positive predictive factors in green. It appears that a worse response to immunotherapy is associated with higher levels of TNF-α and IL-6, a higher proportion of Treg (FOXP3+), or other factors such as IGF-1R mutations. Other signs, on the other hand, indicate to the potential effectiveness of the immunotherapy treatment.

An effort is being made to identify specific biomarkers, which are molecules, cellular, or gene components that can be found in bodily fluids, on the surface of cells, or elsewhere, in order to develop more precise methods of immunotherapy. Disease biomarkers can generally be divided into three categories. Diagnostic biomarkers are substances with the highest sensitivity and specificity for identifying a properly defined nosological unit. Molecules whose levels are directly or indirectly connected to the typical course and severity of the disease are referred to be prognostic biomarkers. These indicators are frequently used to estimate overall survival (OS). The final category includes predictive biomarkers (27–29). Particularly in the treatment of small cell lung cancer, where therapeutic options are sometimes relatively constrained from the start, the identification of reliable predictors is crucial (Figure 4). Predictive markers are primarily useful for correctly assigning a certain immunotherapy type to the right patient cohort, in which the treatment will be most effective, and the benefit will outweigh any potential dangers or side effects. The search for suitable biomarkers among the dozens to hundreds of available choices is ongoing at the moment, but it is yet not possible to identify molecules that are unmistakably appropriate (19, 30, 31).

### Composition of the tumor microenvironment and TIL as potential immunotherapy predictors

**FIGURE 5:**
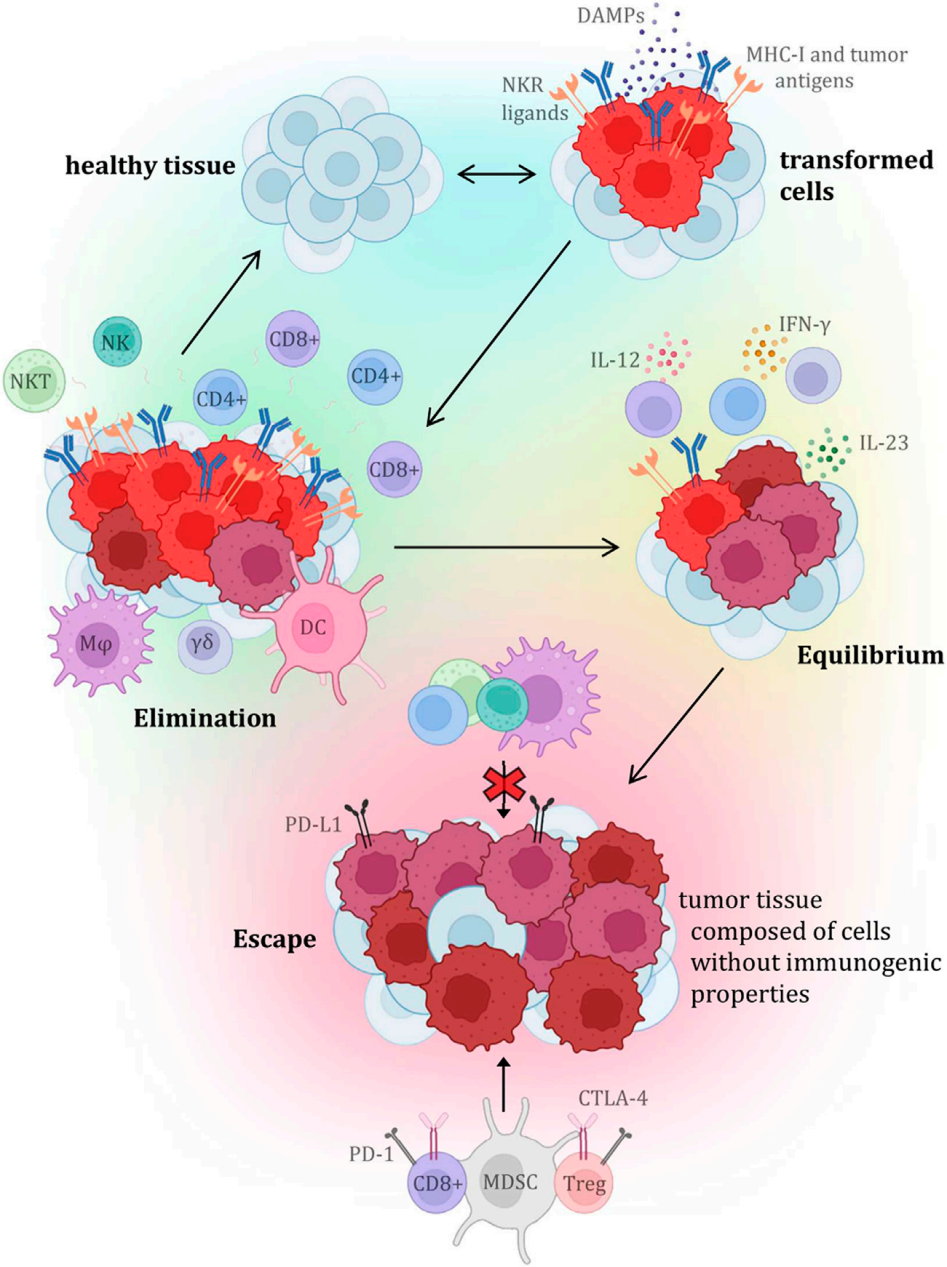
Presents the individual steps of the tumor immunoediting process, which consists of three phases (or three “E”), which are elimination, equilibrium, and escape. Each stage is characterized by specific interactions with variable immune cellularization (primarily with TIL components), and the molecules produced by them (32–39).

Until recently, the main focus of cancer research aimed at the malignant cells themselves, while the importance of other structures in the tumor itself and its immediate surroundings was relatively neglected. Currently, it is already known that many different characteristics, including, for example, standardly assessed aspects such as tumor histopathological morphology, tumor stroma, innervation, or vascularization, have a great influence on the resulting nature of the tumor, its behaviour, response to therapy, and therefore the final prognosis of the malignant disease. Tumor tissue is also known to have antigenic properties and can elicit an immune response, due to the production of altered proteins that can be recognized as dangerous by the host immune system. For this and other reasons, inflammatory cellularization in tumorous, peritumorous and surrounding healthy tissue has been increasingly investigated recently. Over the past 20 years, the general idea of so-called “cancer immunosurveillance” has been further developed, leading to the hypothesis of “tumor immunoediting,” which places more focus on interactions between tumors and the immune system (Figure 5). The original theory that malignant tumors are autonomous cellular diseases with only six biological capabilities (the so-called “hallmarks of cancer”) is gradually being disproved, and research is now focused on the hypothesis that it is a controlled disease involving immune components of the tumor microenvironment itself (and knowledge about the biological capabilities of tumors was also expanded and supplemented). Immune cells have been reported to be able to affect the fate of the tumor according to the three “E” phases—elimination, equilibrium, and escape, by activating innate and adaptive immunity through interactions with tumor cells. One of the most significant and promising areas of research is the ability of human cancers to resist immune destruction. This is related to the increasing focus on the research of so-called tumor-infiltrating lymphocytes (TILs), whose potential is being revealed in terms of prognostic significance as well as a key predictive positive (and in some cases negative) response factor during therapy, especially with immune checkpoint inhibitors (ICIs) (40–43).

**FIGURE 6:**
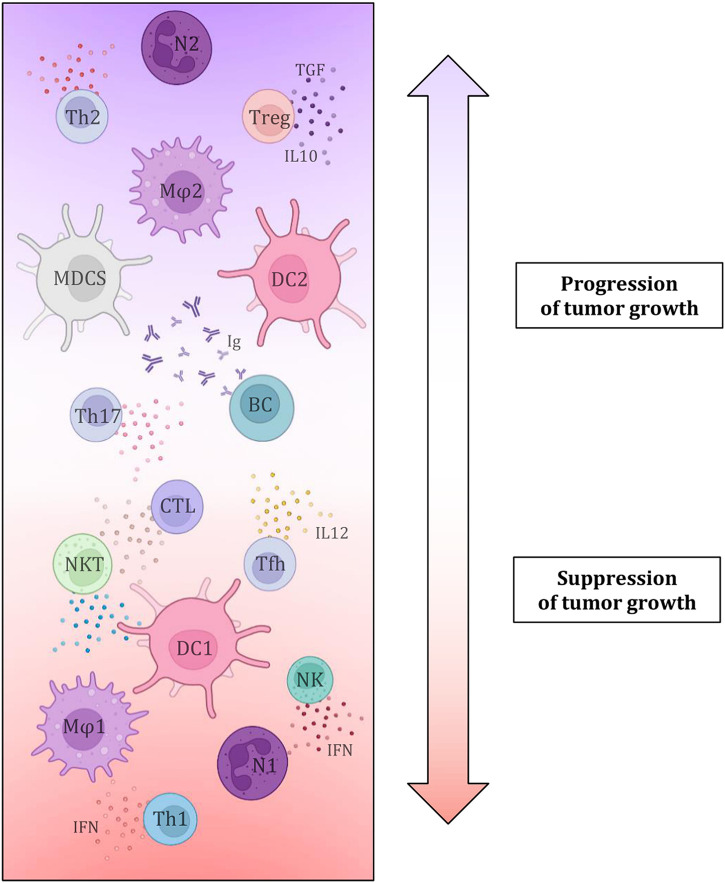
Simply depicts the effect of individual tumor microenvironment components, namely immune cell elements and the substances produced by them, on tumor growth characteristics. This demonstrates conclusively that immune cellularization plays a crucial part in the development of cancer and can, in general, have a favourable impact on the disease (by eliminating tumorous tissue) or, contrary, a negative one (growth potential) (41, 42, 44–50).

The key influence of the composition of the tumor microenvironment and especially the representation of individual TIL components has been debated for a long time, but there is still a lack of conclusive evidence that would permit the introduction of the evaluation of TIL components as a definitive predictive biomarker (Figure 6). Several studies agree that a greater number of tumor-infiltrating and tumor-tissue-related lymphocytes is associated with a more favourable prognosis and higher overall survival, regardless of clinical variables such as TNM staging or operability (51–54). It is assumed that the distribution of CD markers, which reflects the profiles of immune cellularization in the tumor microenvironment, can be a potential predictive biomarker of the effectiveness of immunotherapy in SCLC since the primary mechanism of immunotherapy is precisely the activation of the immune response against tumorous tissue. It is believed that higher levels of the TIL surface biomarkers CD3, CD20, and CD45 are associated with more favourable prognoses and higher survival rates (51–53, 55–57). On the other hand, FOXP3+ TIL (Treg) components are typically viewed as suppressive cells that affect autotolerance and immunological homeostasis, ultimately promoting the growth of the tumorous tissue. Therefore, the interaction between tumor cells and Tregs, which regulate the activity of other T-lymphocytes, particularly cytotoxic T-lymphocytes (CTLs), may have an impact on the effectiveness of immunotherapy in SCLC. A worse prognosis has been documented for SCLC patients with tumor infiltrates that contain larger proportion of FOXP3+ cells. Certain SCLC tumor cell lines induce *de novo* differentiation of functional FOXP3+ Tregs in healthy blood lymphocytes. These are the mechanisms through which SCLC cells suppress immunological responses, which has an impact on immunotherapy efficacy as well as patient survival. However, it is important to note that FOXP3+ TILs are a heterogeneous group that includes not only suppressive elements, but also a non-suppressive population with significant antitumor activity, which necessitates further investigation of the given issue and the definition of a clear opinion on the influence of Treg on tumor progression and treatment with immunotherapeutic methods (51, 52, 57–62). Additionally, it was discovered that patients with a better prognosis had a greater percentage of CD45RO+ memory T-lymphocytes in TIL population. In addition, high numbers of effector T-lymphocytes (especially CTL) were more often found in LD-SCLC (limited-stage disease) compared to ED-SCLC (extensive-stage), and a higher ratio of effector and regulatory T-lymphocytes was also associated with a better outcome. There is a growing list of immune cell markers and specific expression patterns of immunoactive proteins (stimulatory and inhibitory immune checkpoints and cytokines) that need to be researched in the hopes of enhancing the efficacy of immunotherapy since the tumor microenvironment contains not only TILs but also a plethora of other immune cells (dendritic cells, suppressor myeloid cells, macrophages, or neutrophils) along with tumor cellularization and tumor stromal cells (52–56, 60, 63, 64).

### Mutational burden of tumor tissue

Tumor mutation burden (TMB) is defined as the total number of non-synonymous mutations in the tumor genome. Small cell lung cancer is characterized by one of the highest numbers of somatic mutations across malignant tumors, including alterations in DNA repair mechanisms. It is thought to be triggered by long-term smoking, a chronic inflammatory process associated with it, and gene changes brought on by chemicals in cigarette smoke. In general, tumors with higher mutational loads are more likely to produce specific neoantigens that can activate the adaptive immune system’s response. The formation of microsatellite instability (MSI), which results from a lack in DNA mismatch repair genes, is the primary reason for the correlation between a higher number of mutations and the formation of neoepitopes and a stronger response to immunotherapy. Therefore, MSI is a favourable predictive factor of response to ICI therapy, particularly PD-1 blockers. TMB has been shown to be beneficial as an immunotherapy predictor primarily in melanoma and NSCLC, but this suggests that it may also be beneficial for SCLC (51, 52, 65–69). The results of the studies show that TMB is a promising predictive biomarker for checkpoint inhibitor therapy, especially in metastatic SCLC, and the first reliable tissue biomarker for immune checkpoint blockade. Furthermore, patients with high TMB (which represent approximately ¼ of all SCLC cases) have been found to benefit long-term from nivolumab therapy (with or without combination with ipilimumab) (30, 53, 54, 70, 71).

Despite the generally encouraging research findings, standardizing the assessment of cancer tissue mutational load as a specific immunotherapy prognostic factor is currently not entirely feasible in routine clinical practice. The primary issue is that patients with SCLC are given cytological or small biopsy samples, of which quality assessment is quite challenging (which is an obstacle not only for the TMB, but also for the other biomarkers). The increased amount of SCLC-specific necrosis complicates the assessment even more. Therefore, in order to provide reliable standard TMB testing, sufficient tumor tissue must be obtained, ideally through a core cut biopsy, which is not always attainable in all patients (51, 52, 65). In addition, it is advisable to place greater emphasis on the quality rather than just the quantity of the mutations under investigation, as certain mutations are more immunogenic than the others. For instance, indel mutations have a stronger therapeutic response than mismatch mutations. Contrarily, individuals who have mutations in the genes for the IFN-γ signalling pathway receptors (JAK1, JAK2, and APLNR) have a significant treatment resistance (72–76).

### PD-L1 expression

**FIGURE 7:**
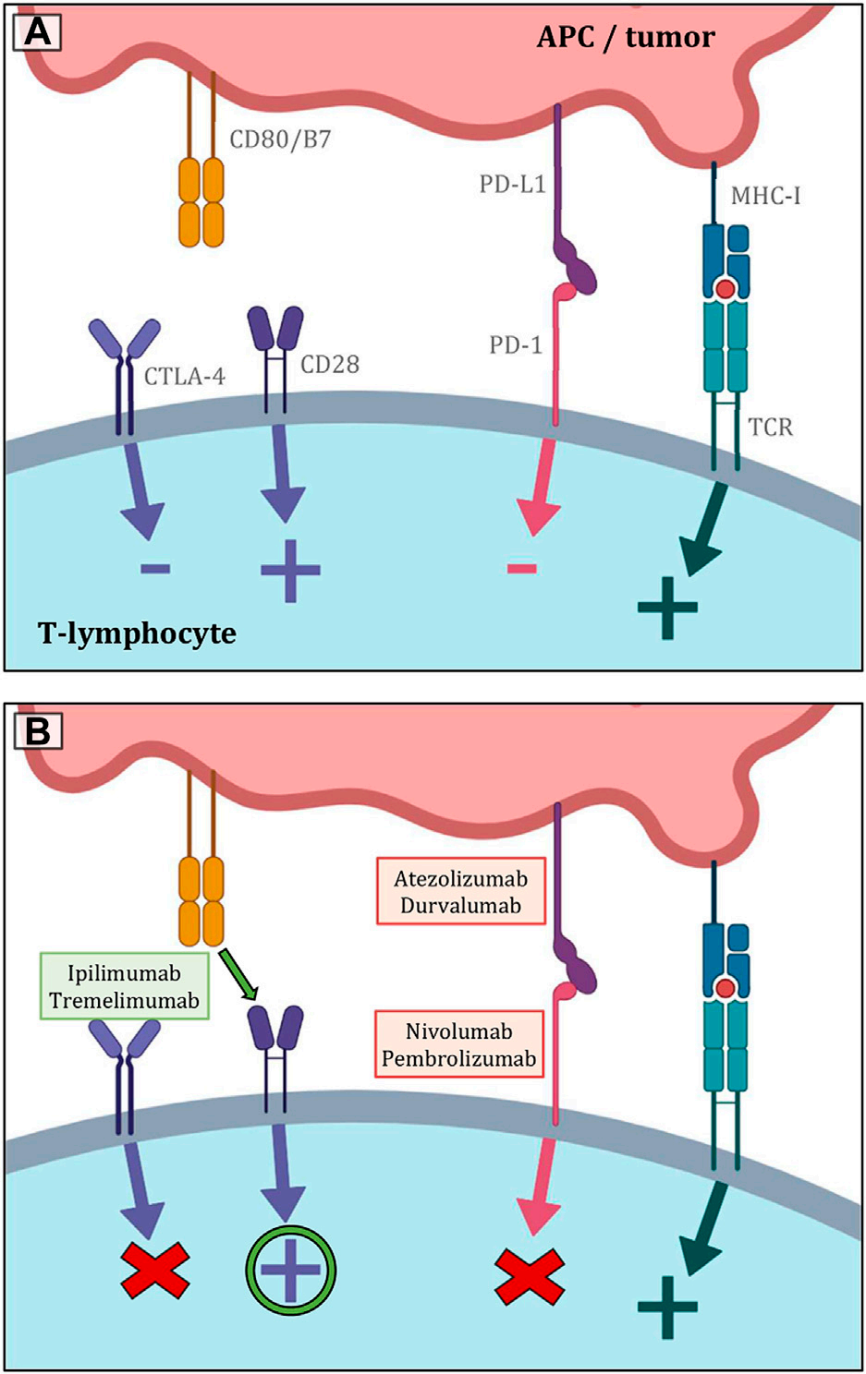
**(A)** Shows the most significant immunological pathways that are crucial for both inducing and inhibiting T-lymphocyte function. The basic stimulatory signal is mediated by the MHC complex during antigen presentation to the TCR receptor. The second signal is co-stimulation by the binding of CD80/B7 to CD28. The primary inhibitory route, in contrast, is represented by the contact between PD-L1 and PD-1. The CTLA-4 receptor then competes with CD28 for CD80/B7 affinity, and the result of its activity is also the inhibition of lymphocyte function. **(B)** Indicates the essential sites of action of the most used immunotherapeutics in the treatment of lung malignancies. Inhibitors of PD-L1 and PD-1 both lessen lymphocytes’ physiological inhibition, which eventually improves anticancer activity. Similar principles govern the blockage of CTLA-4 receptors. As a result, there are more CD80/B7 molecules available to CD28 on the surface of T lymphocytes, stimulating the cell (19–23).

As it is known, the interaction between PD-1 and PD-L1 represents a key mechanism for the escape of tumor cells from immunological elimination, especially by cytotoxic T-lymphocytes (Figure 7). This mechanism basically works in opposition to the increased immunogenicity that comes from a more prominent TMB. In many solid tumors, including NSCLC, the assessment of PD-L1 expression on the surface of tumor cells is recognized to be a highly significant predictive factor for the effectiveness of immunotherapy with PD-1 inhibitors. The primary challenges include the lower prevalence, the fluctuating dynamics of expression, the lack of diagnostic kits and specific antibodies, the heterogeneity of this biomarker’s expression in the context of SCLC, difficulty of obtaining suitable samples, as well as the lack of clear evidence of a correlation between PD-L1 expression and a greater effect of immunotherapeutic procedures. The so-called combined proportion score (CPS), which has a better correlation with treatment outcomes, is also being studied. It is calculated by dividing the total number of viable tumor cells by the number of PD-L1-positive cells, including both tumorous and TIL cells, and multiplying the result by 100. It was also discovered that in SCLC (despite the low prevalence of PD-L1 expression on tumor cells), stromal expression may be more frequent and appears to be a predictive biomarker of therapeutic benefit of pembrolizumab. In contrast, the general hypothesis in the case of nivolumab is that PD-L1 expression cannot be considered a unique criterion for predicting and determining the appropriate patient population that will have a good response to anti-PD-L1 antibody treatment, as the clinical benefit of immunotherapeutic agents has also been observed in patients with PD-L1 negative SCLC (30, 31, 51–53, 65, 71, 77–79). As the heterogeneity of PD-L1 expression is not fully captured by the small sample of tumor tissue collected, circulating tumor cells were examined. However, this type of examination also faces technical problems, such as difficult isolation and subsequent validation. To summarize the data regarding PD-L1 expression in SCLC, this biomarker does not appear to be suitable for patients treated with chemotherapy plus ICI. Furthermore, considering the heterogeneity and plasticity of SCLC, future studies should evaluate other biomarkers than PD-L1 or CPS expression (73, 79–84).

### Molecular subtyping of small cell lung cancer

**FIGURE 8:**
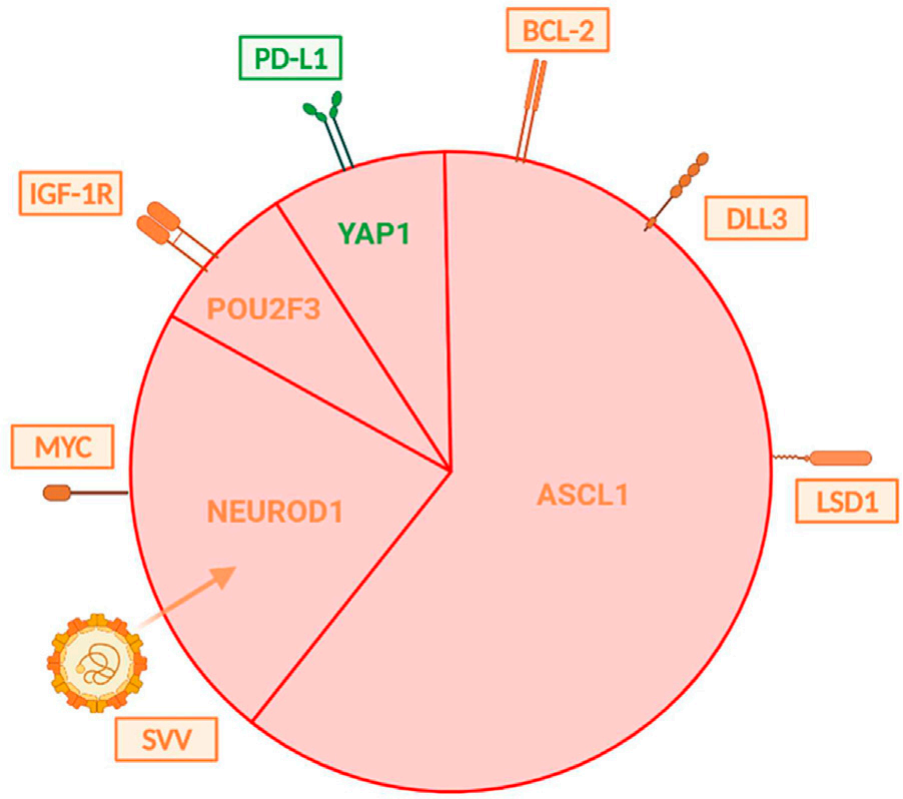
Illustrates the distribution of individual types of SCLC that are distinguished by differences in gene expression. The subtypes vary in their expression of surface molecules, which may play a role in one’s response to appropriate treatment. Immunotherapy targets are shown in green, whereas those for chemotherapy are shown in orange (57, 67).

In contrast to the more personalized approach to NSCLC, SCLCs are still considered a single malignant entity in routine clinical practice. However, gene expression profiling techniques have shown that SCLC displays clinical and molecular heterogeneity, pointing to the existence of four genetically different subtypes of this cancer (Figure 8). They are distinguished based on the relative expression of three or four key transcriptional regulators—ASCL1, NEUROD1, POU2F3, and YAP1 – with ASCL1 and NEUROD1 playing a crucial role in healthy neuroendocrine development. The most significant seems to be the SCLC-I subtype, which is characterized by low expression of ASCL1, NEUROD1 and POU2F3 (and conversely higher expression of YAP1) and also an inflammatory gene profile. This subtype has a higher proportion of cytotoxic CD8^+^ T-lymphocytes in TIL, as well as NK-cells, macrophages, and B-lymphocytes. The expression of HLA, other antigen presentation mechanisms and important molecules such as PD-L1, PD1, CTLA-4, IDO-1, LAG-3 and TIGIT is also more significant. Patients with SCLC-I subtype are thought to have a better therapeutic response to checkpoint inhibitor immunotherapy. Increased expression of BCL-2, DLL3 or LSD1 proteins is described in ASCL1 type, so this could also become a potential therapeutic target. On the other hand, NEUROD1 type is correlated with MYC amplification, suggesting that c-MYC inhibitor therapy may be an option. The enhanced susceptibility of cells to the oncolytic SVV (Seneca Valley virus), which assaults and destroys neuroendocrine tumor cells by cell lysis, is another intriguing characteristic of the NEUROD1 group. For POU2F3, overall therapy could be enhanced by exposure of the tumor to IGF-1R inhibitors, given the higher occurrence of these markers on the cell surface. Regarding the last YAP1 type, a higher expression of PD-L1 was detected compared to the other three mentioned groups, and based on this finding, it is believed that this cohort could show the greatest therapeutic effect in immunotherapy compared to the others. Furthermore, small cell carcinomas are divided into high and low neuroendocrine (NE) groups according to the expression of various neuroendocrine biomarkers (chromogranin, synaptophysin, nerve cell adhesion molecules or gastrin-releasing peptide). Low NE SCLC is characterized by an increased infiltration of immune cells, whereas high NE is characterized by a reduced infiltration, which also indicates different responses to immunotherapy (especially ICI) (19, 52, 57, 85–89).

### The role of the gut microbiome

More research has recently been done on the impact of the gut microbiota on the efficacy of different types of treatment, including immunotherapy. It is a complex ecosystem, the composition of which has a significant impact not only on the development of intestinal tumors but also on the way extraintestinal malignancies respond to chemo- and immunotherapy. In instance, it has been demonstrated that sensitivity to ICI or chemoimmunotherapy in patients with advanced solid tumors, such as melanoma, ovarian, or lung cancer, correlates with the prevalence of particular bacterial species or evidence of an immune response targeting them. One of the microorganisms that can be used as a positive predictor for immunotherapy is *Akkermansia muciniphila*. It is possible that microbial antigens could improve antigen presentation capability and amplify the reactivity of TIL components, even though the underlying mechanisms are yet not fully understood. Additionally, it is hypothesized that gut microorganisms can enter the tumor microenvironment and create chemotactic substances that can promote immune cell migration to tumor locations. This feature of the antitumor immune response demands additional research in light of the considerable preclinical and clinical discoveries of recent years (62, 74, 76, 90, 91).

### Other potential predictors

Other biomarkers that are actively being investigated in an attempt to discover a suitable predictive marker include, for example, the pulmonary immune prognostic index (LIPI; calculated from the serum LDH level and lymphocyte-to-neutrophil ratio), inflammatory biomarkers such as NLR (the neutrophil to lymphocyte ratio) and PLR (the platelet to lymphocyte ratio), galectin-9 expression on TIL or circulating tumor cells. Since it has been established in the past that elevated (or rising) levels of LDH or CRP have a detrimental impact on the disease’s natural course, they are regarded as unfavourable prognostic indicators. In contrast, positive prognostic factors include tumor-expressed organic anion transport polypeptides (OATPs). Further, the density of TCR receptors on the surface of T-lymphocytes or the cfDNA methylation status both show significant promise as prognostic biomarkers. Despite all efforts and the number of potential biomarkers, the possibilities of predicting the response to immunotherapy in SCLC are incredibly dismal and further research with larger cohorts of patients is necessary (52, 53, 73, 92).

## Conclusion

In order to compile an overview of the current state of knowledge about the nature of the tumor process and potential therapeutic options for small cell lung cancer, a literature review was conducted. The significance of potential predictive biomarkers that would allow the appropriate assignment of a certain immunotherapeutic treatment to a suitable cohort of patients was assessed in this context. Although more explored predictive biomarkers are promising, particularly the composition of the tumor microenvironment or the tumor mutation burden, further prospective studies involving more probands are required to confirm their reliability. However, it is clear that this field of study will continue to expand, as developing a reliable method to predict immunotherapy response is a very appealing goal of current medicine and research in the field of targeted cancer therapy.
